# Continuous Monitoring via Tethered Electroencephalography of Spontaneous Recurrent Seizures in Mice

**DOI:** 10.3389/fnbeh.2017.00172

**Published:** 2017-09-14

**Authors:** Na-Ryum Bin, Hongmei Song, Chiping Wu, Marcus Lau, Shuzo Sugita, James H. Eubanks, Liang Zhang

**Affiliations:** ^1^Krembil Research Institute, University Health Network Toronto, ON, Canada; ^2^Department of Physiology, University of Toronto Toronto, ON, Canada; ^3^Department of Neurosurgery, The First Hospital of Jilin University Jilin, China; ^4^Division of Neurosurgery, Department of Surgery, University of Toronto Toronto, ON, Canada; ^5^The Epilepsy Research Program of Ontario Brain Institute Toronto, ON, Canada; ^6^Division of Neurology, Department of Medicine, University of Toronto Toronto, ON, Canada

**Keywords:** convulsion, discharges, EEG, epilepsy, hippocampus, kindling, mice, seizure

## Abstract

We describe here a simple, cost-effective apparatus for continuous tethered electroencephalographic (EEG) monitoring of spontaneous recurrent seizures in mice. We used a small, low torque slip ring as an EEG commutator, mounted the slip ring onto a standard mouse cage and connected rotary wires of the slip ring directly to animal's implanted headset. Modifications were made in the cage to allow for a convenient installation of the slip ring and accommodation of animal ambient activity. We tested the apparatus for hippocampal EEG recordings in adult C57 black mice. Spontaneous recurrent seizures were induced using extended hippocampal kindling (≥95 daily stimulation). Control animals underwent similar hippocampal electrode implantations but no stimulations were given. Combined EEG and webcam monitoring were performed for 24 h daily for 5–9 consecutive days. During the monitoring periods, the animals moved and accessed water and food freely and showed no apparent restriction in ambient cage activities. Ictal-like hippocampal EEG discharges and concurrent convulsive behaviors that are characteristics of spontaneous recurrent seizures were reliably recorded in a majority of the monitoring experiments in extendedly kindled but not in control animals. However, 1–2 rotary wires were disconnected from the implanted headset in some animals after continuous recordings for ≥5 days. The key features and main limitations of our recording apparatus are discussed.

## Introduction

Epilepsy is a chronic neurological disorder characterized by unprovoked or spontaneous recurrent seizures. Electroencephalographic (EEG) recordings are essential for assessing electrographic activity of neuronal populations and detecting seizures in the brain. Tethered EEG (Cavalheiro et al., 1996; Clasadonte et al., 2013; Seo et al., 2013; Leung et al., 2015; Twele et al., 2015) and telemetric EEG (Wither et al., 2012; Puttachary et al., 2015; Gross et al., 2017; Mooney et al., 2017; Scantlebury et al., 2017) have increasingly been used to examine spontaneous recurrent seizures in mouse models. The telemetric recordings offer an advantage of being free of cable-related complications thus are ideal for continuous EEG monitoring for 24 h daily, up to a few weeks. However, in addition to the cost of transmitters along with the size/weight of implantable transmitters that are appropriate for mice, the rate of signal transmission and the number of available bio-potential channels may often restrain telemetric EEG recordings in mouse models. While tethered recordings are not affected by the above factors, tangling of EEG wires often occurs due to animals' ambient movements or convulsive behaviors and may restrict animals' normal behaviors and disrupt EEG signals. Tangled wires therefore present a major challenge for continuous EEG monitoring in freely moving animals without an experimenter's surveillance.

Electrical slip rings, also called rotary electrical interfaces or connectors, are electromechanical devices that transmit electrical signals from a stationary structure to a rotating structure and eliminate wire damage and dangling caused by movable joints. Slip ring commutators or swivels have long been used to prevent or minimize EEG wire tangling in rat models (Micco, 1977; Urban and Alflen, 1981; Andrews and Hutson, 1982; Matsumura et al., 1995; see review of Bertram, 2017). A major challenge in applying slip ring commutators in mouse models is that the force generated by the animal's ambient movements or convulsive behaviors may not be sufficient to turn the majority of commercially available slip rings. A combination of a low torque slip ring with a counterbalanced arm and/or ball bearing device has been used to overcome this hurdle (Bertram, 2017; Watanabe et al., 2017), but with increased complexity in the operation procedure. Slip ring EEG commutators specified for mice are also commercially available (see dragonflyinc.com and trianglebiosystems.com for more information) but they appear to match specific recording apparatus and come at relatively high costs. Moreover, whether these commutators can be used alone for continuous EEG recordings in mice remains to be vigorously tested.

Previous studies have examined spontaneous recurrent seizures in mouse models using continuous tethered EEG and video recordings (Cavalheiro et al., 1996; Clasadonte et al., 2013; Seo et al., 2013; Leung et al., 2015; Twele et al., 2015). EEG signals were reportedly collected 24 h daily for a period of 4 days or a few months. EEG commutators, flexible cables and/or other means, although not detailed in each individual study, might have been used with great success to overcome wire tangling during the prolonged monitoring period. In light of the above information, we attempted to develop a simple, cost-effective slip ring commutator in the hope that our experience may further continuous EEG monitoring of spontaneous recurrent seizures in mouse models.

## Materials and methods

### Materials

The key materials and equipment used in our experiments are listed Table 1. Price information for some of the materials/equipment is presented as per vendors' websites.

**Table 1:** Main materials and equipment used in our experiments.

**Items**	**Products and vendors**	**Price information**
EEG electrode wires	Polyimide-insulated stainless steel wires, outer diameter 0.127 and 0.2 mm; part# 005SW and 008SW, Plastics One, VA, USA	Contact vendor
Male headset pins	Detached from IC sockets (14 pins/piece); part# ED3014-ND, Digikey, Canada	$75 CAD/100 sockets
David Kopf stereotaxic frame	Part# 72-6343, Harvard Apparatus, Quebec, Canada	Contact vendor
Motorized drill	Model 1474 high speed stereotaxic drill, David Kopf Instruments, CA, USA	Contact vendor
Mini drill bit	Part#115-6001, #1 and #1/4, Ball Mill, Carbide, Circuitmedic.com	Contact vendor
Dental acrylic	Part# 1404, Lang Dental Mfg. Co., Inc., Wheeling, IL, USA or Jet denture repair package, Langdental.com/dental products/denture-repair	Contact vendor
Glue (Insta-cure+)	Part# BSI-106C, gap filling, 1/2 oz, Bob Smith Industries, CA, USA	$23 CAD each, Amazon.ca
Plastic weighting boats	Part# 08-732-115 (139 × 139 × 25.4 mm), Fisher Scientific Canada	$242 CAD/500 pieces
Female connection pins	Part# ED1031-ND, Digikey, Canada	$290 CAD/1,000 pins
Cable (24-conductors)	Part# CW6300, Cooner Wires, CA, USA	Contact vendor
Recordings amplifiers	One or two channel microelectrode amplifiers with extended head-stages; model 3,000 or 1,800, A-M systems, WA, USA	$1,030 or $2,310 USD for model 3,000 or 1,800
Digitizer	Digidata 1,400/1,500, Molecular Devices, CA, USA	Contact vendor
Electrophysiology software	pCLAMP, version 10, Molecular Devices, CA, USA	Contact vendor
Webcam	Part# C615, Logitech, Canada	$100 CAD each
Cursor auto-click program	Mini mouse macro program, http://www.turnssoft.com/mini-mouse-macro.html	Free download
Slip ring with 6 wires	Part#1528-1152-ND, Digikey, Canada	$23 CAD each

### Animals

Male C57 black mice (C57BL/6) were obtained from Charles River Laboratory (Senneville St-Constant, Quebec, Canada). All animals were housed in a vivarium that was maintained between 22 and 23°C and under a 12-h light on/off cycle. Food and water were available *ad libitum*. All experimental procedures described below were reviewed and approved by the Animal Care Committee of the University Health Network, in accordance with the guidelines of the Canadian Council on Animal Care. The animals were operated for electrode implantation at 2–3 months of age and allowed to recover for ≥1 week before further experimentations.

### Electrode construction and implantation

**Figure 1:**
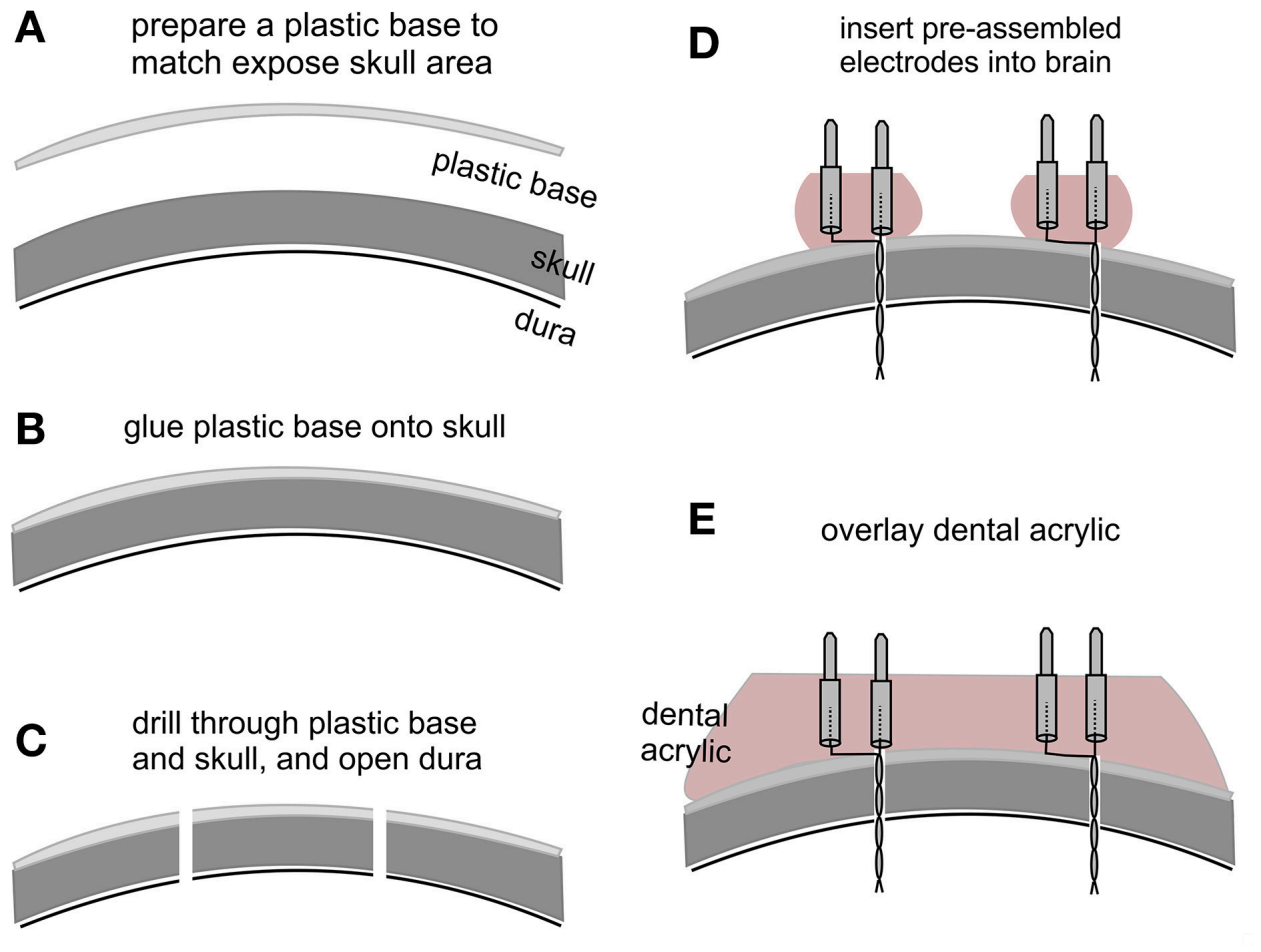
Key steps in electrode implantation. **(A)** A plastic base was cut from a polystyrene weighing dish and its size matched to the exposed skull surface. **(B)** The plastic base was glued onto the skull surface. **(C)** Small holes were drilled through the plastic base and skull, and the dura underneath was opened with a fine needle. **(D)** Pre-assembled bipolar electrodes were inserted into the brain. Note that the electrodes were embedded with dental acrylic to strengthen electrode construct and to facilitate implantation. **(E)** Dental acrylic overlaid onto the plastic base covered the base of inserted electrodes.

All electrodes were made of polyimide-insulated stainless steel wires (outer diameter 0.15 and 0.23 mm; Table 1). We prefer these wires because the polyimide insulation layer is relatively thin (25–30 μm) and strong against mechanical and chemical damage. “Male” pins for implanted headsets were detached from standard IC sockets (Table 1; Figure 1A). We chose these pins for their appropriate length and durability in addition to the wide commercial availability of IC sockets. Twisted bipolar electrodes were constructed as previously described (Jeffrey et al., 2013, 2014), and their lengths matched the targeted brain areas. The base of the pins was embedded with dental acrylic to strengthen the electrode construct and facilitate implantation (Figure 1D).

Electrode implantation was performed similarly as previously described (Wu et al., 2008; Jeffrey et al., 2013). The animal was anesthetized with isoflurane and placed on a stereotaxic frame equipped with two micromanipulators and a manipulator-supported motorized drill (Table 1). Firstly, after skin incision and exposure of the skull, the tip of a mini drill bit was aimed at bregma using the micromanipulator. After determining the position of bregma, the drill bit was moved up but its x-y position corresponding to the bregma remained unchanged. A plastic base matching the size of the skull exposure was cut from the curved portion of a polystyrene weighing dish (0.2 mm thickness; Table 1; Figure 1A). Secondly, the plastic base was glued onto the skull surface using a cyanoacrylate-based glue (Table 1), and x-y coordinates of targeted brain areas were determined and marked on the base (Figure 1B). Thirdly, small holes (≤0.5 mm in diameter) were drilled through the plastic base and the skull, and dura underneath was opened using a fine needle (Figure 1C). For hippocampal kindling/recording experiments, twisted bipolar electrodes were aimed bilaterally to hippocampal CA3 areas (bregma −2.5 mm, lateral 3.0 mm and depth of 2.5 mm; Franklin and Paxinos, 1997). A reference electrode was positioned at a frontal area (bregma +1.0 mm, lateral 1.0 mm, and depth of 0.5 mm). Fourthly, the pre-assembled bipolar electrodes described above were individually inserted into the brain using micromanipulators (Figure 1D). Finally, dental acrylic was overlaid onto the plastic base covering the inserted electrodes (Figure 1E). The above approach is successful likely because the dental acrylic denatures the plastic base and therefore upon hardening bonds the base to the underlying skull to secure the implanted electrodes in place (Wu et al., 2008; Jeffrey et al., 2013).

### Slip ring preparation

**Figure 2:**
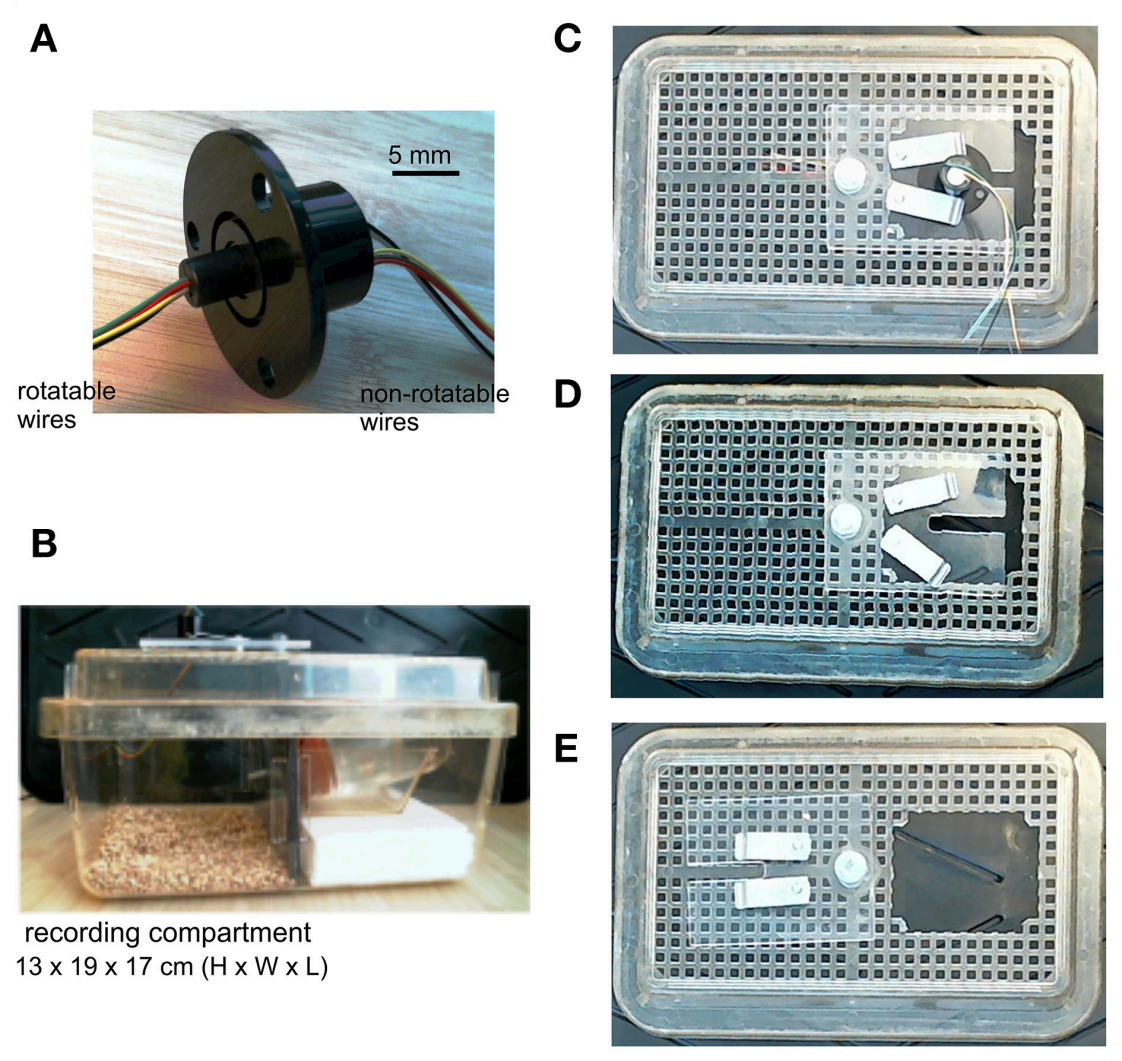
Slip ring and modified mouse cage. **(A)** An image of the slip ring we used. The slip ring has six rotatable and non-rotatable wires and is 22 mm in flange diameter (part# 1528-1152-ND, Digikey, Canada). **(B)** A lateral view of the mouse cage which we modified. The cage was divided by a plastic glass into two compartments, one for animal housing/recordings and another for placing a water bottle. A narrow cut was made in the plastic glass that allowed the outlet of the water bottle to pass through. The slip ring was mounted onto a cage lid. **(C)** Bird's eye views of the cage lid. The lid had an opening of 60 × 50 mm. A plastic glass plate, screwed onto the lid but movable horizontally, was used to cover the opening and to support the flange of the slip ring. Two small metal bars, screwed onto the glass plate but movable horizontally, were used to hold the slip ring by pressing onto its flange. **(D)** The slip ring was dismounted from the plastic glass plate. **(E)** The plastic glass plate was moved away to show the lid opening.

We used a small low torque, 6-wire slip ring as an EEG commutator (Table 1; Figure 2A). Rotatable wires of the slip ring were soldered to female connecting pins (Table 1) that connected directly to animal's implanted headset (Supplementary Video 1) and corresponding non-rotatable wires were connected to EEG amplifier inputs. Five rotatable and non-rotatable wires were used for animals that were implanted with two twisted bipolar electrodes and one reference electrode. Unused wires were cut to reduce noise. Since the original wires of the slip ring were relatively rigid, only one original rotatable wire was used and the others were cut at about 2.5 cm away the slip ring and soldered to soft wires to avoid restriction on cage activities of the animals. The soft wires were detached from a 24-conductor cable (Table 1). The overall length of the rotary wires (from the slip ring to the female connecting pins) was approximately 64 cm, which allowed the animal to move freely in a modified cage (see below). Devices that facilitate slip ring turning, such a counterbalanced bar and/or ball bearing apparatus (Bertram, 2017; Watanabe et al., 2017), were not used in our recording setting.

### Mouse cage modifications

A standard mouse cage (13 × 19 × 29 cm in height, width and length; Microvent model JAG75, Allentown, NJ, USA) was modified for continuous EEG recordings. The cage was divided by a plastic glass into two compartments: one for animal housing/recording and the other compartment for placing a water bottle (Figure 2B). A narrow cut was made in the plastic glass allowing the water bottle outlet to pass through. The width-length of the housing/recording compartment is 17 × 19 cm, which is in keeping with that described by Watanabe et al. (2017). The floor of the recording compartment was covered with bedding materials, and food pellets were provided on the floor. An opening (about 6 × 5 cm) was made in a cage lid to allow for the placement of an animal. A plastic glass plate, screwed on the cage lid but movable horizontally, was used to cover the lid opening and to accommodate the slip ring (Figures 2C–E). The plastic glass plate had a rectangular cut (1 × 4 cm) which supported the flange of the slip ring and allowed rotatable wires of the slip ring to pass through. Two small metal bars, screwed onto the plastic glass plate but movable horizontally, were used to secure the slip ring by pressing onto its flange.

### Setting up continuous EEG-webcam monitoring

Female pins (Table 1) soldered to rotatable wires of the slip ring were used to connect to the mouse's implanted headset pins. We used these female pins because they have inner gaskets and fit tightly to the male headset pins. Tweezers with threaded tips were used to hold the female pins for electrode connection or disconnection. We suggest holding the animal in air by gripping its neck skin with the thumb and forefinger and securing its tail via the little finger and the lower thumb joint but avoid pressing the animal against the hard surface of a table or bench as the latter may cause respiratory suppression. Once an implanted animal was connected to rotatable wires, the animal was placed into the recording compartment through the cage lid opening. The plastic glass plate screwed onto the lid was moved back to cover the lid opening and the slip ring was then secured onto the plastic glass plate by the two small metal bars. The animal was inspected for several minutes to ensure no restriction on its movement. If needed, the bedding level was adjusted to reduce potential restriction on animal behaviors. Afterwards, non-rotatable wires of the slip ring were connected to amplifier's head-stages, which were placed on top of the cage lid. A webcam (Table 1) was placed near the recording compartment of the cage for monitoring of animal motor behaviors.

### EEG data acquisition

One-channel or two-channel microelectrode AC amplifiers with extended head-stages (Am Systems; Table 1) were used for collecting EEG signals as previously described (El-Hayek et al., 2011, 2013; Jeffrey et al., 2014). The input frequency band of these amplifiers was set in a range of 0.1–1,000 Hz and amplification gain at 1,000x. Amplifier's output signals were digitized at 5,000 Hz (Digidata 1400, Molecular Devices; Table 1). Data acquisition, storage, and analysis were done using pCLAMP software (Table 1). To avoid complications of saving and analyzing large data files, EEG and video data were saved every 2 h using the cursor auto-click function of Mini Mouse Macro program (Table 1). A 10-s interval between recording sessions was intentionally given to ensure proper data storage. Data collection was stopped ~30 min daily for the purpose of animal care.

### Extended hippocampal kindling

An extended kindling protocol (Brandt et al., 2004) was adopted to induce spontaneous recurrent seizures in mice. Unilateral hippocampal stimulation was conducted using a Grass stimulator (mode S88) through an isolation unit. Daily electrical stimulation consisted of repetitive square current pulses at 60 Hz for 2 s, with pulse duration of 0.5 ms and intensity at 125% of initial after-discharge threshold as determined using an ascending protocol (Jeffrey et al., 2014). The four animals described below experienced 90–110 daily stimulation prior to continuous EEG-webcam monitoring of spontaneous recurrent seizures.

### Data analysis

EEG and video data were analyzed by an experienced epilepsy researcher and were verified by another experienced researcher. Ictal-like hippocampal EEG discharges were recognized by their large amplitudes (≥2 times of background signals), long durations (≥20 s) and repetitive single or complex spike waveforms. Spontaneous motor seizures were scored using a modified Racine stage (Racine, 1972) for mice (Reddy and Rogawski, 2010). Briefly, stage 0—no response or behavioral arrest; stage 1—chewing or head-nodding; stage 2—chewing and head nodding; stage 3—single or bilateral forelimb clonus; stage 4—bilateral forelimb clonus and rearing; stage 5—rearing and falling with forelimb clonus. Spontaneous recurrent seizures were defined largely by bilateral hippocampal EEG discharges and concurrent stage 3–5 motor seizures. Motor behaviors corresponding to some hippocampal discharges could not be assessed due to errors in video data acquisition. Animals with ≥12 spontaneous seizure events over ≥5 consecutive days were presented below for analysis of spontaneous recurrent seizures.

## Results

### Ambient behaviors and EEG signals observed during continuous monitoring

Continuous EEG-webcam recordings were conducted in 4 mice that exhibited spontaneous recurrent seizures following extended kindling and in 4 non-stimulated control mice. These mice were 9–10 months of age and 24–29 g in body weight prior to the continuous monitoring. EEG and video data were collected ~24 h daily for 5–9 consecutive days on individual animals. During the continuous monitoring periods, the rotary wires of the slip ring were connected directly to animal's implanted headset, and the slip ring turned readily in response to animal movement without involving other devices (such as a counterbalanced bar and/or ball bearing apparatus; Bertram, 2017; Watanabe et al., 2017). In general, the rotary wires were not tangled or temporally twisted for 2–3 turns. The animals under continuous monitoring moved and accessed water and food freely and showed “normal” behaviors such as grooming, rearing or digging (Supplementary Videos 1, 2).

**Figure 3:**
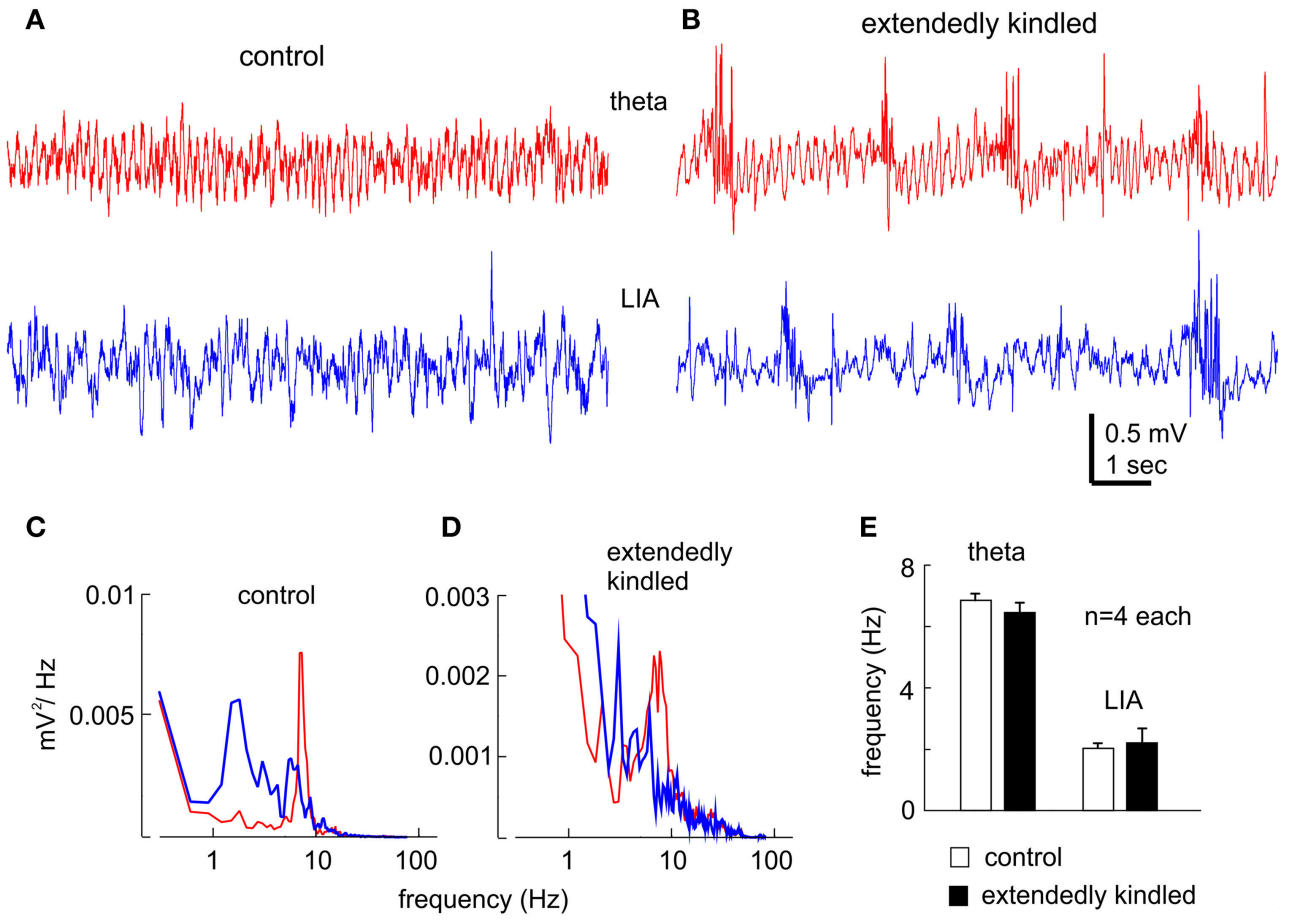
Hippocampal EEG rhythms. **(A,B)** Representative traces collected from a control mouse **(A)** and an extendedly kindled mouse **(B)** showing the theta rhythm (red) and large irregular activity (LIA, blue). Note, interictal spike events in **(B). (C,D)** spectral plots were generated from 15-s data segments including the illustrated signals. **(E)** The main frequencies of the theta rhythm and large irregular activity measured from 4 control mice and 4 extendedly kindled mice. There was no significant group difference in these measures (*p* ≥ 0.3, Student *t*-test).

“Physiological” hippocampal EEG patterns, such as the theta rhythm and the large irregular activity that occur during movement/exploration and immobility/sleep behaviors, respectively (Buzsáki et al., 2003; El-Hayek et al., 2013), were consistently observed in the control mice (Figures 3A,C). The theta rhythm and large irregular activity with similar frequency were also observed from the animals following extended kindling, but these activities were intermingled with aberrant interictal spikes (Figures 3B,D,E).

### Spontaneous recurrent seizures observed during continuous monitoring

**Figure 4:**
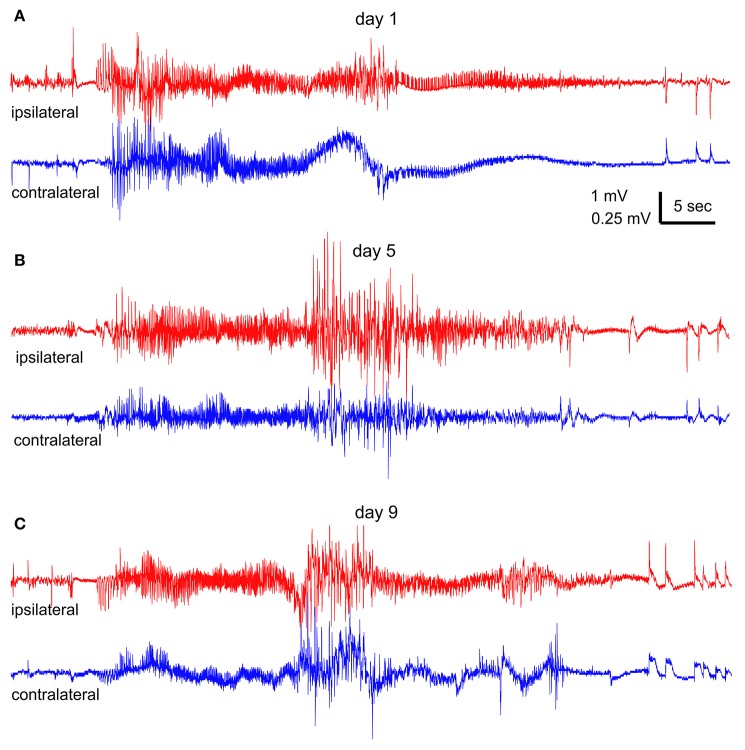
Spontaneous hippocampal EEG discharges. Continuous EEG-webcam monitoring was conducted in an extendedly kindled mouse (#4) over 9 consecutive days. **(A–C)** One episode of bilateral discharges collected at the 1st, 5th, and 9th day, respectively. “Ipsilateral” (red) and “contralateral” (blue) events refereed to the site of hippocampal kindling stimulation. Note the onset time advance of ipsilateral vs. contralateral discharge in **(A)**. An episode of stage-5 or stage-3 motor seizure was observed in correspondence to the discharges in **(B,C)**.

Spontaneous recurrent seizures were observed from all 4 mice that received 90–110 daily hippocampal stimulations and exhibited ≥65 evoked stage 3–5 motor seizures. In contrast, no spontaneous seizure was observed from the 4 control mice. Representative EEG traces recorded from an extensively kindled mouse over a 9-day monitoring period were shown in Figures 4A–C, where a single episode of bilateral hippocampal discharge in the 1st, 5^th^, and 9th day was illustrated. These hippocampal discharges exhibited low-amplitude signals in onset and decreasing signals toward termination. A time advance in ipsilateral discharge onset vs. contralateral discharge onset (ipsilateral and contralateral were in reference to the stimulated site) was evident during the 1st day's recording (Figure 4A) but not in the 5th and 9th days' recordings (Figures 4B,C), implying that these spontaneous discharges initially originated from the stimulated hippocampus. The middle portions of these discharges, particularly those recorded in the 5th and 9th day (Figures 4B,C), were contaminated with large artifacts likely due to the animal's convulsive behaviors.

An episode of stage-5 motor seizure (Supplementary Video 1) was seen in correspondence to the discharges collected on the 5th day (Figure 4B). The motor seizure was evident with head nodding, forelimb clonus, tail erection and falling (a loss of righting reflex) with forelimb clonus and hind-limb extension. An episode of stage-3 motor seizure with head nodding and forelimb clonus was observed in correspondence to the discharges recorded in the 9th day (Figure 4C and Supplementary Video 3). Motor behaviors corresponding to the discharges collected in the 1st day (Figure 4A) could not be determined due to an error in video data collection.

**Figure 5:**
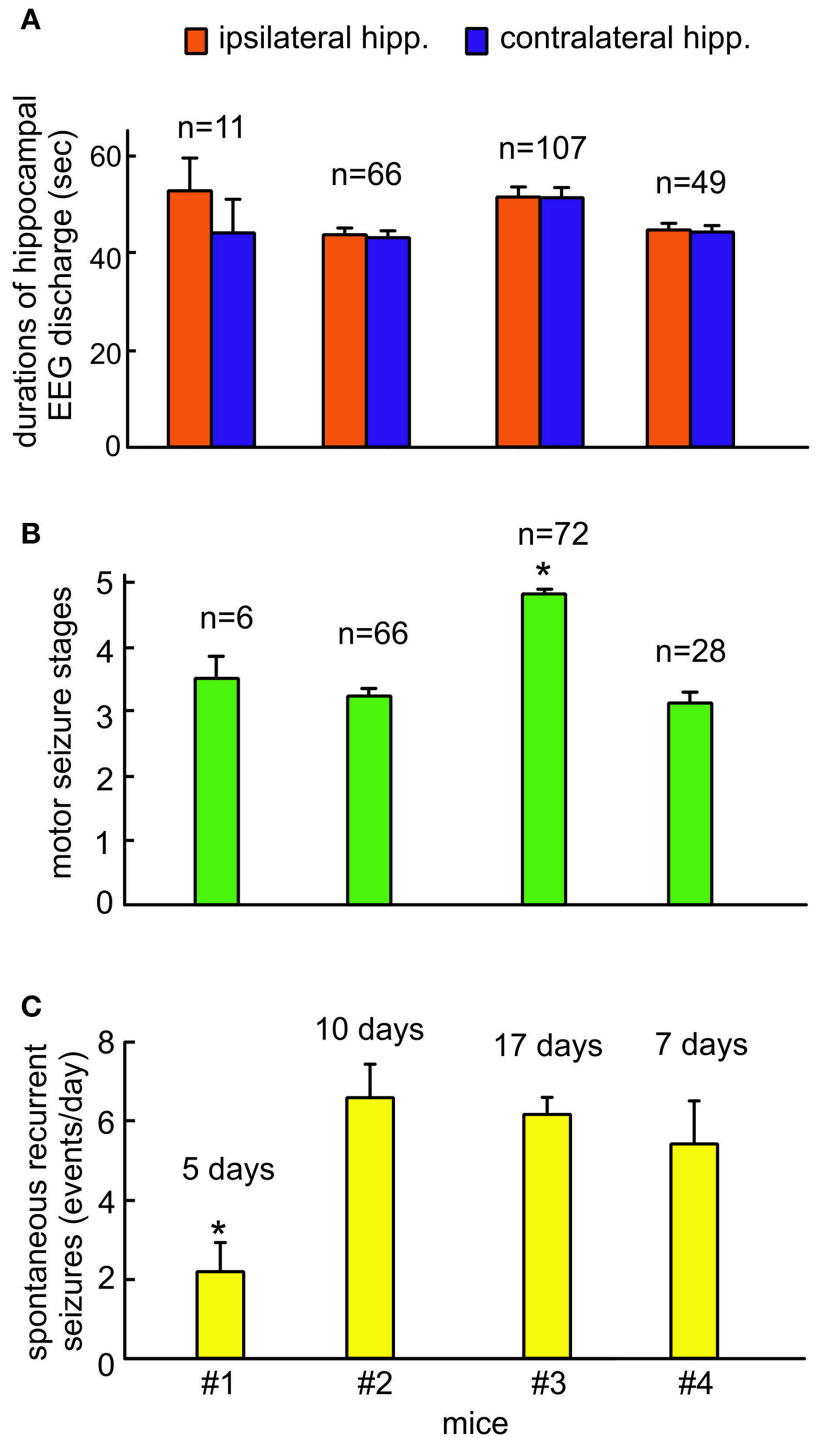
Group measures of hippocampal EEG discharges, motor seizure stages and incidences of spontaneous recurrent seizures. Continuous EEG-webcam monitoring for 5–9 consecutive days was conducted in 4 mice following extended hippocampal kindling. Mice #2 and #3 underwent two monitoring sessions that were 15–30 days apart. Data are presented as means and standard errors of mean. Sample sizes for each measure indicated. **(A)** The durations of ipsilateral and contralateral hippocampal discharges measured from individual animals. **(B)** The stages of motor seizures corresponding to the hippocampal discharges measured from individual animals. Motor seizure scores for some of EEG discharges were not available due to errors in video data acquisition. **(C)** Daily incidences of spontaneous recurrent seizures measured from individual animals. These seizures were determined largely by bilateral hippocampal discharges and concurrent stage 3–5 motor seizures. ^*^Significantly different vs. others, *p* < 0.05, one way ANOVA.

Measurements of EEG discharge durations, motor seizure stages and daily incidences of spontaneous recurrent seizures from the four kindled mice were summarized in Figures 5A–C. The durations of ipsilateral and contralateral hippocampal EEG discharges were not significantly different among the 4 animals (ANOVA, *p* ≥ 0.05), but the stages of motor seizures were significantly greater for the mouse #3 and the incidence of spontaneous recurrent seizures was significantly lower for the mouse #1 relative to other animals (ANOVA, *p* ≤ 0.05). Further works which examine similar spontaneous recurrent seizures in a large cohort of mice will provide clear conclusions behind these individual differences.

## Discussion

### Key features of our recording apparatus

We developed a simple slip ring commutator and operation procedure for continuous EEG monitoring in mice. Specifically, we mounted the slip ring onto the cage lid and connected its rotatory wires directly to the animal's headset. Because there was a relatively short distance between the implanted headset and the slip ring, the force generated by the animal's movement readily turned the slip ring, therefore preventing or minimizing wire tangling. A unique feature of our apparatus is that the slip ring commutator functions alone without extra supporting devices (such as a counterbalanced bar and/or ball bearing apparatus, Bertram, 2017; Watanabe et al., 2017). As such our recording apparatus can be set up in several minutes and no adjustment of extra devices is necessary prior to data collection.

The slip ring commutator and its cage mounting can be managed in a cost-effective manner. The small 6-wire slip ring is expected to be widely available at relatively low costs ($23 CAD each; see Digikey.com/Digikey.ca for more information). The initial cost of assembling a single commutator apparatus, includes a slip ring, female connecting pins, soft cable and materials for cage modification (see details in Table 1 and Section Materials and Methods), is estimated to be ≤ $50 CAD. The cost estimation may vary in different laboratories and regions, but is likely at least several times lower than the costs of commercial EEG commutators. The cost for maintaining the slip ring commutator we described may be minimal since only the female connecting pins and soft cables need to be replaced intermittently.

We validated the slip ring commutator in a mouse model of extended hippocampal kindling and in non-stimulated mice. While these experiments were conducted with a limited number of animals, “physiological” hippocampal EEG signals and ambient cage behaviors were consistently observed from these animals during the continuous monitoring periods. Spontaneous recurrent seizures, featuring bilateral hippocampal discharges and concurrent stage 3–5 convulsions, were reliably recorded from individual kindled animals in a majority of the experiments. To our knowledge, it is presently unclear whether the same slip ring was used in previous studies for continuous tethered EEG recordings in mouse models. Our present works appear to be the first to describe in detail as to how the slip ring can be used as a mouse EEG commutator. We thus anticipate that our works may add to the previously established approaches (Cavalheiro et al., 1996; Clasadonte et al., 2013; Seo et al., 2013; Leung et al., 2015; Twele et al., 2015; Bertram, 2017; Watanabe et al., 2017) and facilitate continuous EEG monitoring in mouse models.

### Limitations, considerations, and potential improvements

We used the female pins to connect the slip ring to implanted headset male pins. These female pins fit tightly to the headset male pins thereby allowing stable data collection during continuous EEG monitoring. However, 1–2 female pins were disconnected from the implanted headset in three animals after 5–9 days of continuous recordings, likely due to wearing of contact between female-male pins. Although, it is convenient to connect the animal to a replacement slip ring, such electrode disconnection disrupts data collection and is a major drawback of our recording apparatus. Other means to stabilize or strengthen the connections between slip ring inputs and implanted headset, such as a harness jacket (Bertram, 2017) or screwed headset caps, should be sought and tested in our model.

In our experiments, EEG signals collected through the slip ring commutator did not appear to be associated with high electronic noise but were prone to movement-related artifacts particularly in those collected over a long monitoring period or through repeated usage of the slip rings. We speculate that contact wearing, resulting in increased contact resistance of the slip ring, may contribute to movement-related artifacts. Because contact wearing is directly related to the total operation time of mechanically operated slip rings and commercial slip rings likely vary in electrical noise levels, it is conceivable to replace the slip rings which have been repeatedly used for a considerable amount of time. Such replacement may not be a major financial burden for individual laboratories since the slip ring is available at a low cost relative to commercially available EEG commutators.

The spontaneous recurrent seizures we observed from the mouse model of extended hippocampal kindling were featured with moderate convulsive behaviors such as head nodding, body shaking, forelimb clonus, rearing, and/or falling. Our recording apparatus could overcome wire tangling related to these moderate convulsive seizures and reliably captured concurrent hippocampal discharges. We anticipate that our recording apparatus may also be feasible for continuous monitoring of similar seizure activity in other mouse models such as the late-onset seizures observed in adult mice following neonatal hypoxia-ischemia episodes (Peng et al., 2015). However, our recording apparatus is unlikely to overcome wire tangling caused by vigorous convulsive behaviors such as fast running, jumping, and/or barrel-rolling observed from mouse models of early-onset post-ischemic seizures (El-Hayek et al., 2011; Wang et al., 2015; Wu et al., 2015). Additionally, since adult mice were tested in our present experiments, it remains yet to be determined as to whether the recording apparatus described here is suitable for continuous EEG monitoring in younger mice with lower body weights.

We monitored spontaneous recurrent seizures via combined EEG and webcam recordings. EEG signals were recorded in a wide frequency band of 0.1–1,000 Hz and digitized at 5,000 Hz, but video data were captured at a temporal resolution of 25–30 frames per second and not synchronized with the EEG data. In addition, our apparatus does not have the capacity for concurrent EEG/electromyogram recordings and drug infusion (Watanabe et al., 2017). Further improvements of our apparatus in these aspects are needed for better assessment of spontaneous recurrent seizures in mouse models.

## Author contributions

NB: Experimentation and manuscript writing; HS: Experimentation and data analysis; ML: experimentation; CW: Experimental design and experimentation; SS: Experimental design and data discussion; JE: Experimental design and data discussion; LZ: Experimental design, data discussion and manuscript writing.

### Conflict of interest statement

The authors declare that the research was conducted in the absence of any commercial or financial relationships that could be construed as a potential conflict of interest.

## Supplementary material

Supplementary Video 1 Moving and exploring behaviors observed from a control mouse. Continuous EEG-webcam monitoring over 5 consecutive days was conducted in a control mouse. A representative video segment was collected in the 4th days of the monitoring. Note that the animal moved freely in cage together with exploring, grooming, and digging behaviors.

Supplementary Video 2 Drinking and eating behaviors observed from a mouse following extended hippocampal kindling. Continuous EEG-webcam monitoring over 9 consecutive days was conducted in a mouse following extended hippocampal kindling. A representative video segment was collected in the 8th days of the monitoring, showing animal's drinking, eating, and grooming behaviors.

Supplementary Video 3 Spontaneous motor seizures observed from a mouse following extended hippocampal kindling. Continuous EEG-webcam monitoring over 9 consecutive days was conducted in a mouse (same animal as shown in Supplementary Video 2) following extended hippocampal kindling. An episode of stage 5 motor seizure was captured at the 5th day of monitoring, which was in correspondence to the hippocampal EEG discharges illustrated in Figure 3B. The motor seizure started at about the 28th second of video time and featured with head nodding, forelimb clonus, tail erection and fall with forelimb clonus and hind-limb extension. The female pins connecting to animal's implanted electrodes were visible at the beginning of the video.
